# Epigallocatechin-3-gallate protects cardiomyocytes from hypoxia-reoxygenation damage via raising autophagy related 4C expression

**DOI:** 10.1080/21655979.2021.1996018

**Published:** 2021-12-02

**Authors:** Ping Liu, Jin Huang, Wanzhen Mei, Xingfang Zeng, Cheng Wang, Chuan Wen, Jing Xu

**Affiliations:** a Clinical Nursing Teaching and Research Section, The Second Xiangya Hospital, Central South University, Changsha, China; b Department of Pediatric Neurology and Cardiovasology, Children’s Medical Center, The Second Xiangya Hospital, Central South University, Changsha, China; c Department of Pediatric Hematology and Oncology, Children’s Medical Center, the Second Xiangya Hospital, Central South University, Changsha, China

**Keywords:** Myocardial ischemia/reperfusion, cardiomyocyte hypoxia/reoxygenation, epigallocatechin-3-gallate, H9c2 cells, reactive oxygen species, autophagy related 4C

## Abstract

Myocardial ischemia/reperfusion (I/R) injury is a serious issue during the therapy of myocardial infarction. Herein, we explored the beneficial influence of Epigallocatechin-3-gallate (EGCG) on hypoxia/reoxygenation (H/R)-stimulated cardiomyocyte H9c2 cells damage, along with possible internal molecular mechanism related autophagy related 4C (ATG4C). H9c2 cells were subjected to H/R stimulation and/or EGCG treatment. ATG4C mRNA expression was measured via q-PCR assay. ATG4C overexpression plasmid (OE-ATG4C) was transfected to arise ATG4C level. Cell viability, apoptosis, reactive oxygen species (ROS) production, ATP level were tested via CCK-8 assay, Annexin V-FITC/PI staining, DCFH-DA staining and ATP Assay Kit, respectively. Western blotting was performed to test Cleaved-caspase 3, Cleaved-caspase 9, cytochrome C, and LC3B protein levels. H/R stimulation resulted in H9c2 cell viability loss, promoted cell apoptosis, and ROS overproduction, as well as lowered ATP level in cells. EGCG treatment alleviated H/R-resulted H9c2 cell viability loss, cell apoptosis, ROS overproduction, and reduction of ATP level. Moreover, H/R stimulation reduced the ATG4C expression in H9c2 cells, while EGCG raised the ATG4C expression. Overexpression of ATG4C strengthened the beneficial influence of EGCG on H/R-stimulated H9c2 cell viability, apoptosis and ROS production. Besides, ATG4C overexpression weakened the H/R-stimulated H9c2 cell autophagy via reducing LC3B II/I expression. EGCG exerted beneficial influence on H/R-stimulated cardiomyocytes, which protected cardiomyocytes from H/R-stimulated viability loss, apoptosis, and ROS overproduction via enhancing ATG4C expression.

## Introduction

As is known to all of us, adequate blood supply is essential for the heart to perform normal physiological functions [1]. When the heart undergoes ischemia, the delivery of oxygen, glucose, and other nutrients to the myocardium is reduced, which will lead to the mitochondrial oxidative phosphorylation process of cardiomyocytes be blocked and the contractile function cannot be performed normally [2]. Acute and persistent ischemia will result in irreversible damage to the heart, and even cause myocardial infarction (MI), a major reason for mortality of people with coronary artery disorder [3,4]. Besides, as the main therapeutic strategy for ischemia, blood reperfusion also can damage cardiomyocytes, termed ‘reperfusion-injury’ [5,6]. Studying the molecular mechanism related to myocardial ischemia/reperfusion (I/R) injury and searching strategies methods that can weaken I/R-resulted cardiomyocytes damage is believed to have great value for prevention and therapy of coronary artery disorder.

Green tea is a popular beverage. In recent years, the negative correlation between green tea drinking and the incidence of cardiovascular diseases has been widely reported [7,8]. Epigallocatechin-3-gallate (EGCG, CAS number: 989–51-5) is the main active and water-soluble component of green tea [9]. As a catechin monomer, EGCG has a special stereochemical structure and possesses very strong antioxidant activity [10]. Lots of literature reported that EGCG exhibited beneficial activity in treating coronary artery disorder [11,12]. More importantly, EGCG was discovered to have protective activity on I/R-resulted damage in many tissues, such as the kidney [13], brain [14], skeletal muscle [15], and heart [16]. Salameh *et al*. [17] discovered that EGCG could alleviate I/R-resulted damage of isolated perfused rabbit hearts. Townsend *et al*. [18] revealed that EGCG could lessen I/R-resulted cardiomyocytes apoptosis *in vitro* and *in vivo*. More studies are still demanded to further exploring the myocardial beneficial activity of EGCG subjecting to I/R stimulation, which will provide experimental basis for EGCG as a new drug for coronary artery disorder therapy.

H9c2 cell line is a subclone of the cloned cell line of BD1X rat embryonic heart tissue, which is often used to investigate cardiomyocyte damage after I/R or other stimulation [19,20]. Previous literature reported that H9c2 cells subjecting to hypoxia/reoxygenation (H/R) stimulation *in vitro* can simulate the damage of cardiomyocytes caused by I/R *in vivo* [21]. We proposed that EGCG could alleviate H/R-caused H9c2 cell damage. In the current research, following H/R stimulation, the possible beneficial function of EGCG treatment on H9c2 cell viability, apoptosis, reactive oxygen species (ROS) production, ATP generation was further investigated. Besides, whether autophagy related 4C (ATG4C) takes part in this process was analyzed. We think that further understanding the possible beneficial influence of EGCG on H/R-stimulated H9c2 cells will be helpful for the prevention and therapy of myocardial I/R injury.

## Materials and method

### Cell culture and H/R stimulation

H9c2 cells were provided by Stem Cell Bank, Chinese Academy of Science (Shanghai, China), and growth in DMEM (Sigma-Aldrich, MO, USA) replenishing with 10% (v/v) fetal bovine serum (FBS, Invitrogen, CA, USA) and 1% (v/v) Penicillin-Streptomycin solution (Procell Life Science & Technology Co., Ltd. Wuhan, China).

To simulate cardiomyocytes’ damage after I/R stimulation, H9c2 cells were grown in an anoxic chamber with 5% CO_2_ and 95% N_2_ for 6 h and then grown in a normal chamber with 95% air and 5% CO_2_ for 12 h.

### Preparation of EGCG

EGCG (Sigma-Aldrich, purity > 95%, CatLog number: E4143) was dissolved in distilled water to 100 mg/L and saved at −4°C. Before experiments, phosphate buffer saline (PBS) was used to dilute EGCG solution to 2, 4, 8, 16, 32, or 64 mg/L. For H9c2 cells both stimulated by H/R and treated by EGCG, EGCG was pre-added into the culture medium for 24 h, after that, the culture medium was changed and cells were received H/R stimulation.

### Cell transfection

A full-length sequence of ATG4C was inserted into pcDNA3.0 plasmid (GeneChem Corporation, Shanghai, China) to construct OE-ATG4C, which was transfected in H9c2 cells via Lipofectamine^TM^ 2000 Reagent (Invitrogen) [22]. After 48 h, the transfection efficiency of OE-ATG4C was tested via qPCR assay. For H9c2 cells both subjected to OE-ATG4C transfection and H/R or EGCG treatment, OE-ATG4C was pre-transfected for 48 h, after that, the cells received EGCG treatment and/or H/R stimulation.

### qRT-PCR assay

qRT-PCR assay was conducted similarly as stated in the earlier literature [23]. Total RNAs were detached from H9c2 cells using Trizol Reagent (Takara Biotechnology, Beijing, China). Then, 2 μg RNAs were acted as temple to composite cDNA via Bestar^TM^ qPCR RT Kit (DBI Bioscience, Shanghai, China). Real-time PCR was carried out using Bestar^TM^ qPCR MasterMix (DBI Bioscience) with reaction condition: 2 min at 95°C, 40 cycle of 20 s at 94°C, 20 s at 58°C, and 20 s at 72°C. GAPDH expression was served as an internal control. The primer sequences were as follows: 5ʹ-ACCCCAACAATTTCTCTGAAGG-3ʹ (ATG4C-F), 5ʹ-GTCCATACCAATCTCCTGCTTTT-3ʹ (ATG4C-R), 5ʹ-TGTTCGTCATGGGTGTGAAC-3ʹ (GAPDH-F), and 5ʹ-ATGGCATGGACTGTGGTCAT-3ʹ (GAPDH-R). Data were analyzed by the 2^−ΔΔCt^ method.

### Test of cell viability

After relevant stimulation and/or treatment, the viability of H9c2 cells was tested via cell counting Kit-8 (CCK-8) assay (Beyotime Biotechnology, Shanghai, China) [22]. H9c2 cells were cultivated in a 96-well plate with 1 × 10^4^ cells/well for 24 or 48 h. Then, 10 μL CCK-8 solution was replenished in each well for 2 h. Subsequently, the absorbance value at 450 nm (OD_450 nm_) of each well was measured through a Micro-plate reader (Bio-Tek Inc., MO, USA).

### Measurement of cell apoptosis

After relevant stimulation and/or treatment, cell apoptosis was tested via Annexin V-FITC/PI Apoptosis Detection Kit (Yeasen Biotechnology, Co., Ltd. Shanghai, China) [24]. A total of 3 × 10^4^ H9c2 cells were cultivated in a 24-well plate for 24 h. Then, cells in each group were gathered, rinsed using PBS, and dyed with 5 μL Annexin V-FITC and 10 μL PI for 15 min protected from light. The apoptosis rate was measured via a flow cytometer (BD Biosciences, NJ, USA).

### Detection of ROS production

2ʹ,7ʹ-dichlorofluorescein-diacetate (DCFH-DA) is a fluorescent probe that can transform non-fluorescent DCFH in cells [25]. ROS can oxidize DCFH to form DCF that has high fluorescence. So, the fluorescence intensity of DCF in cells can reflect the intracellular ROS concentration. After relevant stimulation and/or treatment, 3 × 10^4^ cells H9c2 cells were cultivated in a 24-well plate for 6 h. Then, cells in each group were collected, rinsed using PBS and stained with 10 μM DCFH-DA (Sigma-Aldrich) for 20 min protected from light. The ROS generation was detected via a flow cytometer.

### Assessment of ATP concentration

After relevant stimulation and/or treatment, the ATP concentration in H9c2 cells was tested via ATP Assay Kit (Beyotime Biotechnology) [26]. A total of 3 × 10^4^ H9c2 cells were cultivated in a 24-well plate for 24 h. Then, cells were collected, mixed with cell lysis buffer for 10 min and centrifuged at 12,000 g at 4°C for 5 min. The ATP concentration in supernatant was tested through incubating with 100 μL kit solution and recorded using Luminometer (Maxwell Technologies Inc., CA, USA).

### Western blotting

Total proteins were detached using RIPA Lysis Buffer (Beyotime Biotechnology). Western blotting was performed similarly as earlier literature reported [22]. Primary antibodies, including cleaved-caspase 3 (#9664), cleaved-caspase 9 (#9507), Cytochrome C (#4280), LC3B (#2775), β-actin (#3700), and GAPDH (#8884) were all obtained from Cell Signaling Technology (MA, USA). Secondary antibodies, including HRP Goat anti-Mouse IgG (BA1051) or HRP Goat anti-Rabbit IgG (BA1054) were provided by Boster Biological Technology (Wuhan, China). Bands of protein were visualized via enhanced chemiluminescence technique and the intensities of bands were analyzed via Image-Pro Plus 6.0 software.

### Statistical analysis

GraphPad Prism 9.0 software was conducted for statistical analysis. Data were displayed as mean ± standard deviation (SD) from three repeated experiments. One-way ANOVA was performed for calculating *P* values with a significance level of *P* < 0.05.

## Results

### EGCG suppressed H/R-caused H9c2 cell viability loss and apoptosis

**Figure 1.**
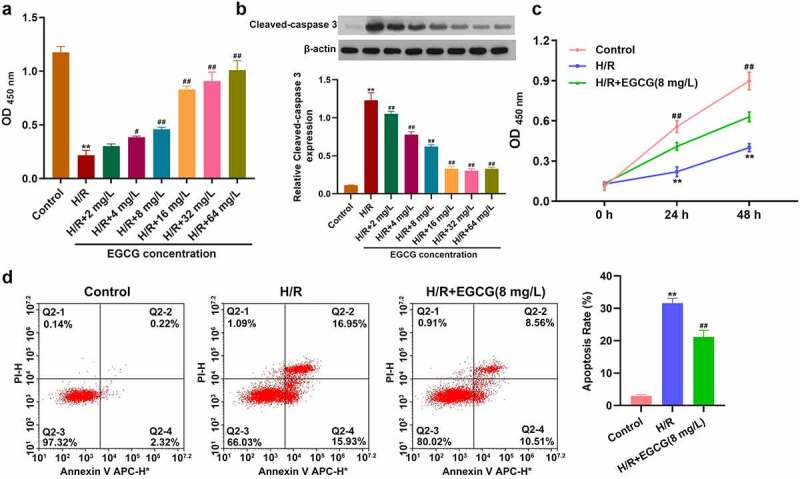
EGCG suppressed H/R-resulted H9c2 cell viability reduction and apoptosis

Firstly, whether EGCG could exert protective effect on H/R-stimulated H9c2 cells was explored. Following H/R stimulation and 2, 4, 8, 16, 32, or 64 mg/L EGCG treatment, H9c2 cell viability was tested. Figure 1a displays that H/R stimulation significantly lowered the H9c2 cell viability (*P* < 0.01), while 4, 8, 16, 32, or 64 mg/L EGCG treatment notably raised the H9c2 cell viability (*P* < 0.05 or *P* < 0.01). Figure 1b shows that H/R stimulation noticeably raised the Cleaved-caspase 3 expression in H9c2 cells (*P* < 0.01). Relative to H/R group, the Cleaved-caspase 3 expressions were remarkably reduced in H/R + 2, 4, 8, 16, 32, or 64 mg/L EGCG group (*P* < 0.01). 8 mg/L EGCG was selected for follow-up experiments. Moreover, Figure 1c illustrates that H/R stimulation reduced the viability of H9c2 cells in time-dependent manner (*P* < 0.01), while 8 mg/L EGCG treatment alleviated the reduction of H9c2 cell viability in both 24 and 48 h (*P* < 0.01). Figure 1d presents that H/R stimulation notably promoted H9c2 cell apoptosis (*P* < 0.01), but 8 mg/L EGCG treatment weakened the H/R-resulted H9c2 cell apoptosis (*P* < 0.01). These outcomes represented suggested that EGCG suppressed H/R-resulted H9c2 cell viability reduction and apoptosis.

Following H/R stimulation and 2, 4, 8, 16, 32, or 64 mg/L EGCG treatment, H9c2 cell viability was checked by CCK-8 assay (a) and cleaved-caspase 3 in cells was tested via western blotting (b). Following H/R stimulation and 8 mg/L EGCG treatment, the H9c2 cell viability was checked via CCK-8 assay (c) and cell apoptosis was tested via Annexin V-FITC/PI staining and flow cytometer (d). **P < 0.01 *vs*. Control group; ^#^*P* < 0.05, ^##^*P* < 0.01 *vs*. H/R group.

### EGCG lessened H/R-resulted ROS overproduction, ATP loss and mitochondrial-dependent apoptosis

**Figure 2.**
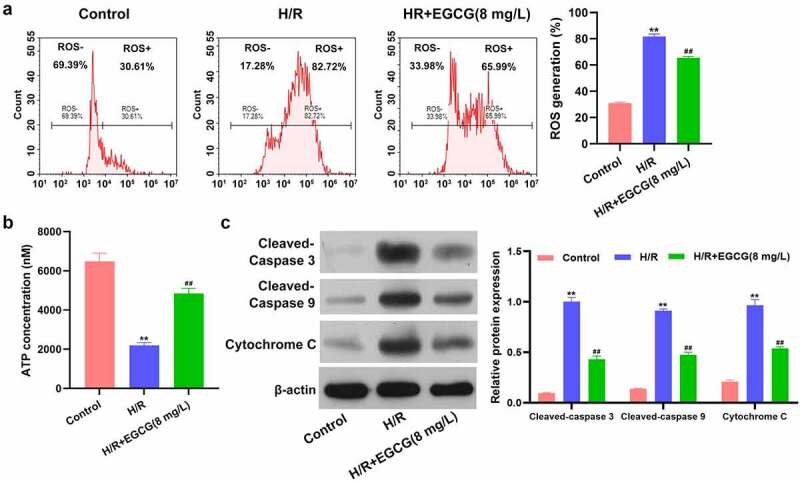
EGCG lessened H/R-resulted ROS generation, ATP loss and mitochondrial damage

Then, the influences of H/R stimulation and EGCG treatment on ROS production and ATP concentrations in H9c2 cells were detected. Figure 2a displays that H/R stimulation notably raised the ROS production in H9c2 cells (*P* < 0.01). Compared to H/R group, the ROS generation was decreased in H/R + 8 mg/L EGCG group (*P* < 0.01). Moreover, Figure 2b illustrates that H/R stimulation remarkably reduced the ATP concentration in H9c2 cells (*P* < 0.01), while 8 mg/L EGCG treatment notably attenuated the H/R-resulted reduction of ATP concentration (*P* < 0.01). Besides, H/R stimulation elevated the cleaved-Caspase 3, cleaved-Caspase 9 and cytochrome C expressions in H9c2 cells (Figure 2c, * P*< 0.01). Relative to H/R group, the cleaved-Caspase 3, cleaved-Caspase 9, and cytochrome C expressions were lowered in H/R + 8 mg/L EGCG group (*P* < 0.01). These above outcomes represented that EGCG lessened H/R-resulted ROS overproduction, ATP loss, and mitochondrial-dependent apoptosis.

Following H/R stimulation and 8 mg/L EGCG treatment, the ROS production of H9c2 cells was measured via DCFH-DA staining and flow cytometer (a), the ATP concentration in H9c2 cells was tested by ATP Assay Kit (b), and the cleaved-Caspase 3, cleaved-Caspase 9, and cytochrome C expressions were evaluated via western blotting (c). **P < 0.01 *vs*. Control group; ^##^*P* < 0.01 *vs*. H/R group.

### EGCG reversed H/R-resulted reduction of ATG4C expression in H9c2 cells

**Figure 3.**
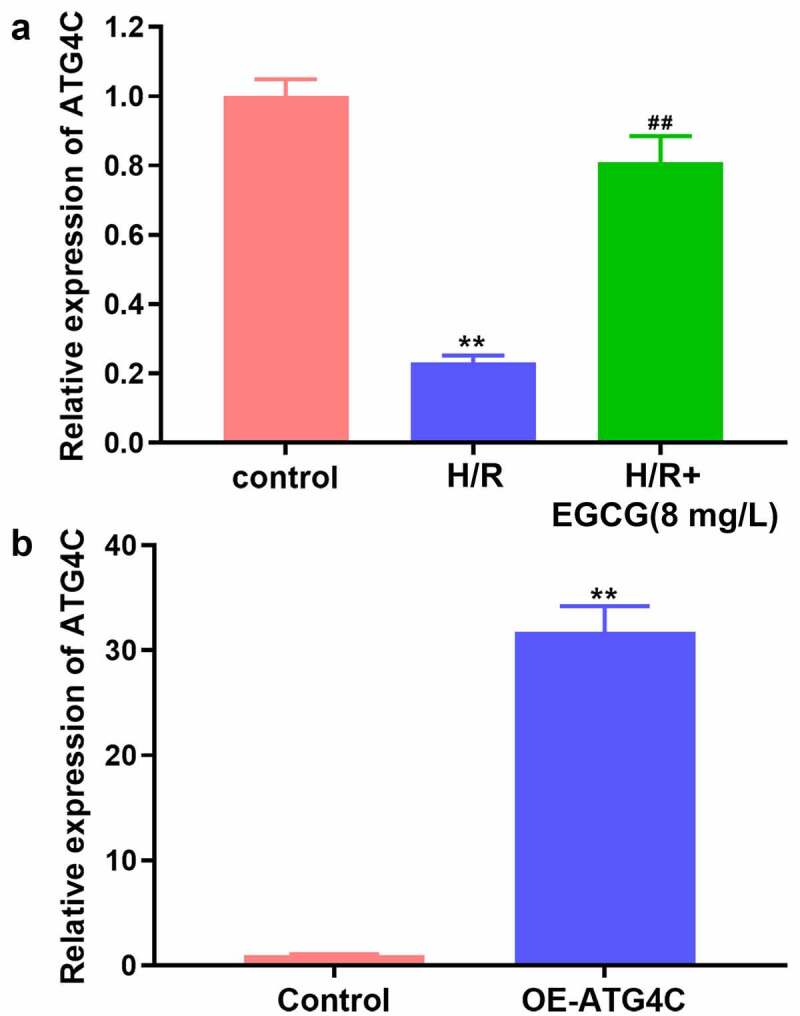
EGCG reversed H/R-resulted reduction of ATG4C expression in H9c2 cells

Previous literature reported that ATG4C expression was down-regulated in myocardial tissue of mice with myocardial I/R damage [27], which implied that ATG4C might be a potential regulator involving in the cardiomyocyte’s functions. Herein, the influence of H/R stimulation and EGCG treatment on ATG4C expression in H9c2 cells was tested. In consistent with earlier research, in this study, H/R stimulation also significantly down-regulated the ATG4C mRNA expression in H9c2 cells (Figure 3a, * P*< 0.01). However, 8 mg/L EGCG treatment notably attenuated the H/R-resulted decrease of ATG4C mRNA expression (*P* < 0.01). To overexpress ATG4C in H9c2 cells, OE-ATG4C was transfected in H9c2 cells. Figure 3b displays that OE-ATG4C transfection remarkably enhanced the ATG4C mRNA expression (*P* < 0.01). These outcomes implied that EGCG reversed the H/R-resulted H9c2 cell damage possible via raising ATG4C expression.

(a) H9c2 cells were stimulated by H/R and treated by 8 mg/L EGCG, the ATG4C mRNA expression was examined via q-PCR assay. (b) H9c2 cells was transfected OE-ATG4C, the ATG4C mRNA expression was examined via q-PCR assay.

### Overexpression of ATG4C strengthened the beneficial activity of EGCG on H/R-treated H9c2 cells

**Figure 4.**
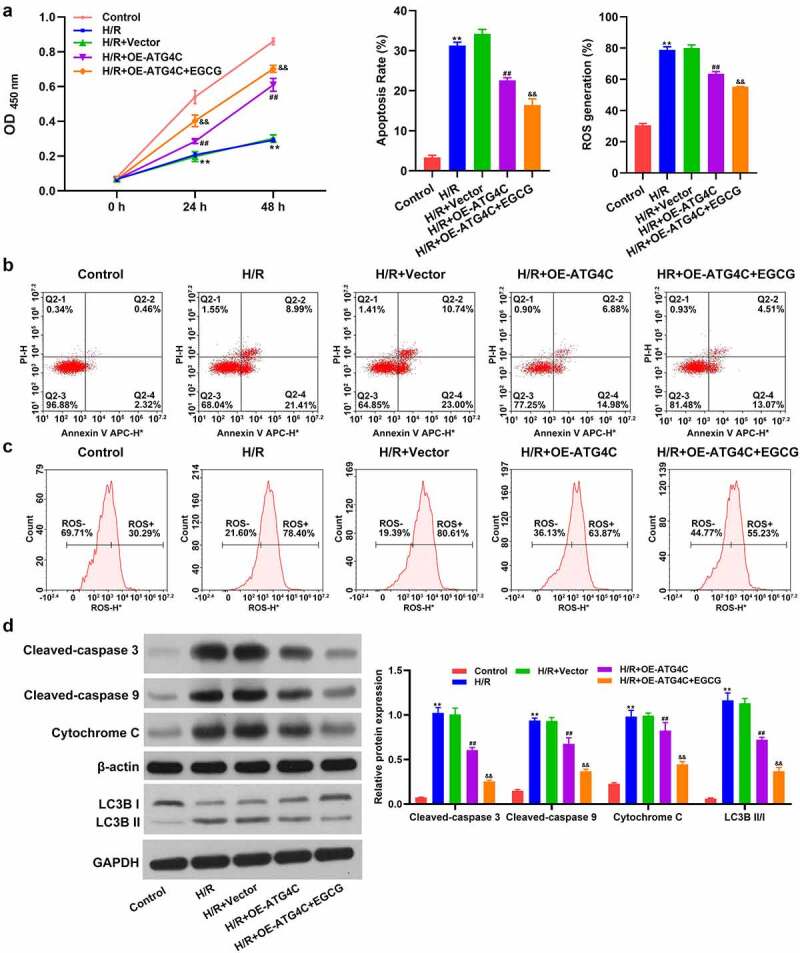
Overexpression of ATG4C strengthened the beneficial influence of EGCG on H/R-treated H9c2 cells

Finally, whether ATG4C took part in the influence of EGCG on H/R-stimulated H9c2 cell viability, ROS production and mitochondrial-dependent apoptosis were explored. Figure 4a displays that similar to 8 mg/L EGCG treatment, OE-ATG4C transfection also partially reversed the H/R-resulted H9c2 cell viability loss (*P* < 0.01). Relative to H/R+ OE-ATG4C group, the cell viability was hoisted in H/R+ OE-ATG4C+EGCG group (*P* < 0.01). Moreover, Figures 4b and c show that OE-ATG4C transfection also notably declined the H/R-resulted H9c2 cell apoptosis and ROS production (*P* < 0.01). 8 mg/L EGCG treatment further reduced the H/R-resulted H9c2 cell apoptosis and ROS production, as evidenced by the reductions of apoptotic rate (%) and ROS generation (%) in H/R+ OE-ATG4C+EGCG group. Figure 4d illustrates that similar to 8 mg/L EGCG treatment, OE-ATG4C noticeably lowered the cleaved-Caspase 3, cleaved-Caspase 9, and cytochrome C protein expressions in H9c2 cells (*P* < 0.01). Relative to H/R+ OE-ATG4C group, the expressions of these three proteins were lowered in H/R+ OE-ATG4C+EGCG group (*P* < 0.01). Besides, the LC3B II/I protein expression in H9c2 cells was notably increased after H/R stimulation (*P* < 0.01). OE-ATG4C transfection reduced the H/R-resulted increase of LC3B II/I protein expression in H9c2 cells (*P* < 0.01). Relative to H/R+ OE-ATG4C group, the LC3B II/I protein expression was further lowered in H/R+ OE-ATG4C+EGCG group (*P* < 0.01). These outcomes represented that ATG4C participated in the modulation of H9c2 cell autophagy, which overexpression strengthened the beneficial activity of EGCG on H/R-treated H9c2 cells.

Following H/R stimulation, OE-ATG4C transfection and 8 mg/L EGCG treatment, H9c2 cell viability was checked via CCK-8 assay (a), cell apoptosis = was tested via Annexin V-FITC/PI staining and flow cytometer (b), ROS production was measured via DCFH-DA staining and flow cytometer (c), and cleaved-Caspase 3, cleaved-Caspase 9, cytochrome C, and LC3B expressions were evaluated via western blotting (d). **P < 0.01 *vs*. Control group; ^##^*P* < 0.01 *vs*. H/R+ Vector group; ^&&^*P* < 0.01 *vs*. H/R+ OE-ATG4C group.

## Discussion

I/R damage is a phenomenon in which myocardial tissue damage is intensified after the blood reperfusion to the ischemia myocardial tissue [5]. This has become a generally concerned clinical problem by researchers. Reduction of cardiomyocytes damage caused by I/R is considered to be effective in the prevention and therapy of coronary artery disorder [28,29]. Multiple literature have reported that cardiomyocyte apoptosis is the main form of myocardial injury [30,31]. It is revealed that decreased cardiomyocyte apoptosis can protect the heart from I/R injury [30]. For example, long non-coding RNA myocardial infarction-associated transcript (MIAT) is discovered to weaken reduce I/R injury via reducing cardiomyocytes apoptosis [32]. Polo-like kinase 1 (PLK1) is demonstrated to alleviate I/R-caused myocardial apoptosis via promoting mitophagy [6]. Previous studies demonstrated that EGCG could inhibit cardiomyocytes apoptosis in I/R-stimulated rat model [18,33]. Moreover, EGCG also could alleviate H9c2 cell apoptosis caused by H/R stimulation [34]. In consistent with previous research, we also discovered that EGCG could reduce H/R-resulted H9c2 cell apoptosis. As one of the main marker proteins of mitochondrial apoptosis [35], the Cleaved-caspase 3 expression were lowered by EGCG treatment, which were accompanied with the decreased Cleaved-caspase 9 and Cytochrome C expressions, other two main proteins of mitochondrial apoptosis [35]. These discoveries signified that EGCG unleashed protective influence on H/R-stimulated cardiomyocytes at least via reducing mitochondrial-dependent apoptosis.

The equilibrium among ROS production and consumption is important to maintain normal cell physiological activities [36]. There is a complete pathway of oxidation-antioxidant mechanism in cells to keep the ROS level in a stable range [36]. Previous literature reported that the production of ROS exceeded the neutralizing ability of myocytes for ROS is a main reason for I/R damage [37]. Excessive ROS can trigger lipid peroxidation, inactive multiple intracellular proteins and break DNA strands [38]. Furthermore, excessive ROS generation is also associated with cardiomyocyte apoptosis [39]. Considering that EGCG possessed an outstanding anti-oxidation activity [10], whether EGCG exerted cardio-protective effect during I/R via modulating ROS level was explored. We revealed that H/R stimulation notably raised the ROS production in H9c2 cells, while EGCG treatment lowered the ROS production. These discoveries signified that EGCG-mediated reduction of ROS also contribute to its cardio-protective effect.

Any activity of the cells requires the support of energy. As the universal ‘currency’ for energy transfer in cells, the stability of ATP levels is also crucial for cardiomyocytes [40]. Earlier literature discovered that apart from cell apoptosis and ROS overproduction, I/R also can cause enhancement of intracellular calcium ions, lead to overload of mitochondrial calcium ions, followed by damage of mitochondrial structure and reduction of ATP production l [37]. Cell apoptosis, overproduction of ROS, and reduction of ATP level are closely related [40]. In recent years, lots of compounds, such as vitexin [41] and Ginsenoside Re [42], are discovered to alleviate I/R damage of cardiomyocytes via improving ATP content. Qin *et al*. [43], discovered that EGCG played cerebral protective activity after I/R injury via elevating ATP level. In this study, we found that EGCG raised the ATP level in H/R-treated H9c2 cells. These discoveries signified that EGCG-mediated elevation of ATP level is also beneficial to its cardio-protective effect.

ATG4C, a member of autophagy-related gene family, is a cysteine peptidase that take part in the modulation of LC3 lipidation and de-lipidation, as well as C-terminal peptide cleavage of ATG8, in the cell autophagy process [44,45]. Sun *et al*., demonstrated that ATG4C expression was reduced in mice with myocardial I/R injury [27]. What is more, EGCG was discovered to modulate autophagy in multiple cells [46]. For example, Li *et al*. [47], revealed that EGCG could promote autophagy in endotoxin-stimulated macrophages. Holczer *et al*. [48], reported that EGCG promoted autophagy-dependent survival through modulating mTOR-AMPK pathways. Herein, we discovered that H/R stimulation cut down the ATG4C expression in H9c2 cells, while EGCG treatment raised the ATG4C expression. More importantly, overexpression of ATG4C strengthened the beneficial influence of EGCG on H/R-stimulated H9c2 cell viability loss, mitochondrial-dependent apoptosis, and ROS overproduction. Besides, ATG4C overexpression attenuated the H/R-stimulated H9c2 cell autophagy via reducing LC3B II/I expression. EGCG treatment strengthened the influence of ATG4C overexpression on LC3B II/I expression in H/R-stimulated H9c2 cells. Considering that both autophagy and apoptosis have happened in cardiomyocytes after I/R stimulation [49]. Some literature reported that enhancing cardiomyocytes autophagy could reduce cardiomyocytes apoptosis caused by I/R [50,51]. Some studies discovered that a several of compound or protein could alleviate both I/R-stimulated cardiomyocytes apoptosis and autophagy simultaneously [52,53]. In this research, we revealed that ATG4C overexpression weakened H/R-stimulated H9c2 cell autophagy and EGCG treatment strengthened the effect of ATG4C overexpression, which hinted that EGCG could alleviate I/R-caused cardiomyocytes apoptosis and autophagy at the same time. These discoveries signified that EGCG weakened H/R-resulted H9c2 cell damage could be achieved through raising ATG4C expression.

## Conclusion

Taken together, this research confirmed the beneficial activity of EGCG on H/R-stimulated cardiomyocytes. EGCG declined H/R-resulted cardiomyocytes viability loss, apoptosis, and ROS over-production via enhancing ATG4C expression. Considering that ATG4C is a key protein involving in cell autophagy, further investigations concerning the modulatory influence of EGCG on cardiomyocytes autophagy during H/R stimulation are still needed in the future.

## Article highlights

1. EGCG suppresses H/R-resulted H9c2 cell viability loss and apoptosis;

2. EGCG reduces H/R-resulted ROS over-generation and ATP loss;

3. EGCG reverses H/R-resulted reduction of ATG4C expression in H9c2 cells;

4. ATG4C overexpression strengthens the influence of EGCG on H/R-treated H9c2 cells;

5. ATG4C overexpression alleviates H/R-resulted H9c2 cell autophagy.
